# Exercise Is Medicine: How Do We Implement It?

**DOI:** 10.3390/nu15143164

**Published:** 2023-07-17

**Authors:** Aliya Khasanova, Tara M. Henagan

**Affiliations:** 1 Department of Family Medicine, Baton Rouge General Family Health Center, Baton Rouge, LA 70806, USA; bashkortka@gmail.com; 2 Department of Family Medicine, Baton Rouge General Hospital, Baton Rouge, LA 70808, USA

**Keywords:** exercise, physical activity, obesity, type 2 diabetes, cardiovascular disease, cancer, depression, heart failure, US physical activity guidelines, clinical practice

## Abstract

Exercise is well known to have beneficial effects on various disease states. In this paper, we broadly describe the fundamental concepts that are shared among various disease states, including obesity, type 2 diabetes (T2D), cardiovascular disease (CVD), heart failure (HF), cancer, and psychological well-being, and the beneficial effects of exercise training within these concepts. We highlight issues involved in implementing exercise recommendations and describe the potential impacts and challenges to medical professionals and patients. Problems are identified and discussed with respect to the future roles of professionals in the current built environment with its limited infrastructure to support current physical activity recommendations.

## 1. Exercise and Energy Expenditure

Exercise has been shown to improve outcomes in a variety of disease states, as well as in preventing disease [1]. The effects of exercise for the prevention and treatment of disease may be due to its effects on energy regulation [1]. It is well known that energy regulation is dependent on the intensity of exercise training [2], with lower-intensity endurance training activating aerobic respiratory pathways and high-intensity aerobic training and weight training activating more anaerobic respiratory pathways [3,4]. For example, marathon training increases beta oxidation of fatty acids in addition to the glycolytic catabolism of carbohydrates stored in the form of glycogen as well as those carbohydrates ingested during the training session [5,6]. Conversely, high-intensity interval training (HIIT) activates creatine kinase to break down glycogen quickly and increases glycolysis for glucose catabolism, sparing fatty acids [4,6]. Although aerobic exercise is known to increase fatty acid oxidation during an exercise bout [7], anaerobic exercise has been shown to increase the capacity of fatty acid oxidation in the post-exercise interval as well as throughout the day by increasing the basal metabolic rate and muscle mass [8,9]. Increased muscle mass contributes to increases in the thermic effect of exercise as well as increases in the basal metabolic rate [8,9]. Thus, anaerobic exercise may also lead to more long-term fatty acid oxidation outside of the actual exercise bout. Whether aerobic or anaerobic in nature, exercise training increases overall total energy expenditure. Although energy balance in humans is much more complex than simply considering energy intake vs. energy expended, in theory and in the most simplistic terms, exercise training in the absence of changes in energy intake leads to negative energy balance and weight loss.

## 2. Exercise and Obesity and T2D

One of the largest crises that the United States currently faces is the obesity epidemic, which is closely related to the increased incidence and prevalence of CVD and T2D. In the most simplistic of theories and terms, obesity is a state of positive energy balance, where energy intake exceeds energy expenditure. Given the beneficial effects of both aerobic and anaerobic exercise on energy metabolism, the potential of exercise training to prevent and treat obesity via its ability to increase fatty acid oxidation and energy expenditure is clear [7,10]. In turn, weight loss, even as little as 5% of one’s body weight, is known to improve insulin sensitivity and T2D [11]. Additionally, skeletal muscle contraction itself is known to activate GLUT4 receptors to increase glucose uptake into skeletal muscle, independent of insulin [12]. Thus, unique to exercise as a form of medicine in the treatment of T2D, exercise itself, without changes in body weight, can improve insulin sensitivity and ameliorate T2D. Obesity is a state that is also defined by chronic inflammation [13]. In fact, it has been shown that chronic inflammation may be one of the causative factors in determining an obese state or in maintaining obesity. Chronic inflammation is also linked to insulin resistance and T2D, and alleviating chronic inflammation has been shown to improve insulin sensitivity and reverse T2D [14]. Exercise training, both aerobic and anaerobic training, is known to acutely increase inflammation, largely due to microenvironmental insults that occur during an exercise bout [15]. However, in the recovery period following exercise, this acute increase in inflammatory markers, such as cytokines, leads to recruitment and activation of anti-inflammatory cells and pathways [16,17]. Activation of these anti-inflammatory pathways leads to long-term downregulation of chronic inflammation in response to repeated exercise bouts [17,18,19]. Given the long-term exercise-induced reductions in chronic inflammation, exercise training may play a large role in preventing and treating diseases associated with chronic inflammation.

## 3. Exercise and CVD and HF

CVD is marked by increased levels of inflammation and atherosclerosis and is also prevented and treated with exercise [20,21]. Atherosclerosis is marked by decreased vascular compliance, which plays a role in hypertension and CVD [22,23]. Aortic stiffness or lack of compliance has been inversely correlated with low density lipoprotein (LDL) levels and positively correlated with the amount of energy expended during physical activity [22], and exercise training itself has been shown to improve arterial compliance [23]. Similar to its effects in T2D, exercise also increases fatty acid oxidation and decreases LDL cholesterol, the substrate known to largely contribute to decreased arterial compliance, atherosclerosis, and increased CVD risk [7,24,25,26]. As previously discussed, beyond its ability to alter fatty acid and cholesterol metabolism, exercise training also decreases chronic inflammation. Chronic inflammation is a contributing event in the onset and progression of atherosclerosis and CVD [21]. Collectively, exercise training improves vascular compliance and vascular health and decreases CVD risk [27,28]. In addition to the effects of chronic inflammation in the pathogenesis of CVD, studies have also found that inflammation contributes to HF. Indeed, inflammatory markers have been positively correlated with the progression, prognosis, and severity of HF [29], and physical activity levels are inversely related to HF risk and incidence [30,31]. The benefits of exercise training for HF risk reduction and prevention may be attributed to its effects on cardiac muscle and remodeling and also on chronic inflammation [32,33].

## 4. Exercise and Cancer

Increased physical activity levels and exercise training have been linked to cancer prevention, with longer time spent in sedentary behaviors being associated with cancer risk and with increased physical activity levels and exercise positively impacting breast, colon, and ovarian cancers and multiple myeloma [34]. Similar to its effects in the prevention and treatment of other disease states, exercise training may act to prevent cancer by modulating chronic inflammation, a contributing factor in cancer onset and progression [35]. Exercise may also regulate cellular growth and cancer by altering levels of estrogen and growth factors that are involved in uncontrolled cellular proliferation [36]. Interestingly, obesity and insulin resistance are also risk factors that appear to be involved in cancer onset and that impact cancer risk [37,38,39]. Metabolic adaptations that occur during obesity, including increased levels of fatty acids and blood glucose levels, increase the available substrates for cancer cell growth and metabolism [40,41]. The increased energy expended during and following exercise alters the microenvironment by increasing fatty acid and glucose uptake and utilization by skeletal muscle and other peripheral tissues, limiting available resources for cancer cells and inhibiting tumor growth [42].

## 5. Psychological Benefits of Exercise

In addition to its beneficial effects on energy metabolism, exercise training has been shown to improve psychological well-being [43]. For example, exercise training has been shown to improve depression and mental health with exercise-induced psychological improvements similar to those seen in response to antidepressant therapies [44]. Serum concentrations of endogenous opioids, in particular β-endorphin and β-lipotrophin, are elevated during exercise or training [45]. Elevated β-endorphins during exercise have been linked to several psychological and physiological changes, including mood state changes and exercise-induced euphoria, decreases in pain perception, and positive alterations in stress response hormones, including cortisol [46,47,48]. Exercise-induced improvements in depression have also been associated with exercise-induced improvements in chronic inflammation, as depression is associated with chronic inflammation, and exercise improves depression and decreases chronic inflammation [49,50]. Physical activity levels have also been associated with cognitive functioning and memory, with individuals with lower physical activity levels having a higher risk for developing cognitive impairment and dementia later in life [51]. Interestingly, aging alone is associated with increased levels of chronic inflammation [52]. In individuals with cognitive impairment, exercise training has been shown to increase cognitive function [53], and in individuals with Alzheimer’s disease, it increases memory performance [54,55]. Indeed, both aerobic and anaerobic exercise training have been shown to improve memory, and these improvements have been associated with decreased inflammation [56,57,58].

## 6. Exercise Protects Future Generations

Exercise training not only has the ability to alter the course of disease in a specific individual but also has the ability to prevent disease in future generations. Exercise not only improves outcomes in obesity, T2D, CVD, etc., but also alters the epigenome. Exercise impacts HDAC activity [59], histone acetylation [60], histone methylation [61], histone phosphorylation [62], DNA methylation [63,64], nucleosome positioning [64,65], and, it has been postulated, lactylation [66]. Furthermore, exercise-induced epigenetic alterations have been shown to be inherited by offspring [67]. Interestingly, exercise during pregnancy and paternal exercise in the preconception period both improve glucose tolerance in offspring [68,69], and exercise training attenuates insulin resistance in offspring fed a high-fat diet [70]. Additionally, the effects of maternal exercise may abolish any detrimental metabolic effects of obesity on offspring via epigenetic mechanisms [44]. While it is clear that exercise training affects epigenetic modifications and these modifications are heritable and improve obesity- and T2DM-related outcomes [59,60,61,62,63,64,65,66,67,68,69,70], studies on the potential heritable effects of exercise on CVD, stroke, cancer, etc., are lacking. However, given the pleotropic effects of exercise in preventing and improving T2D and obesity and its ability to alter the epigenome [10,12,59,60,61,62,63,64,65,66,67,68,69,70], it is possible that the effects of maternal and paternal exercise are far-reaching and affect offspring outcomes with respect to other chronic disease states.

## 7. Clinical Recommendations for Implementing Exercise and Barriers

Current clinical recommendations in the Unites States are stratified by age [71]. It is recommended that US adults participate in 150–300 min of moderate-intensity or 75–150 min of vigorous-intensity aerobic physical activity per week, with this activity being spread out throughout the week [71]. In addition, more recent recommendations have acknowledged the health benefits of anaerobic training in the form of resistance training and incorporated the guideline that US adults should participate in muscle-strengthening activities of moderate or greater intensity that involve all major muscle groups on two or more days a week [71]. For most clinic patients, incorporating physical activity and exercise guidelines into their everyday lives is difficult. There are many barriers to doing so in many parts of the US, including access to a safe place to perform exercise training, access to weight training equipment or gyms, cost of memberships at facilities where equipment is available, travel distance and time to and from facilities, working knowledge of how to start and maintain an exercise routine to continue to reap the health benefits of exercise training, how to optimize recovery from training, etc. [72,73]. Although medical doctors are aware of the Physical Activity Guidelines for Americans and many regularly counsel their patients on such guidelines, most medical doctors are not trained or knowledgeable regarding how to create an exercise plan, especially weight training plans based on the various goals of this exercise modality, how to counsel patients on post-exercise recovery periods, how to counsel patients on pre- and post-exercise nutrition, what constitutes “moderate” and “vigorous” exercise, the best exercise modalities for individuals given physical limitations, etc. [74].

## 8. Conclusions

**Figure 1:**
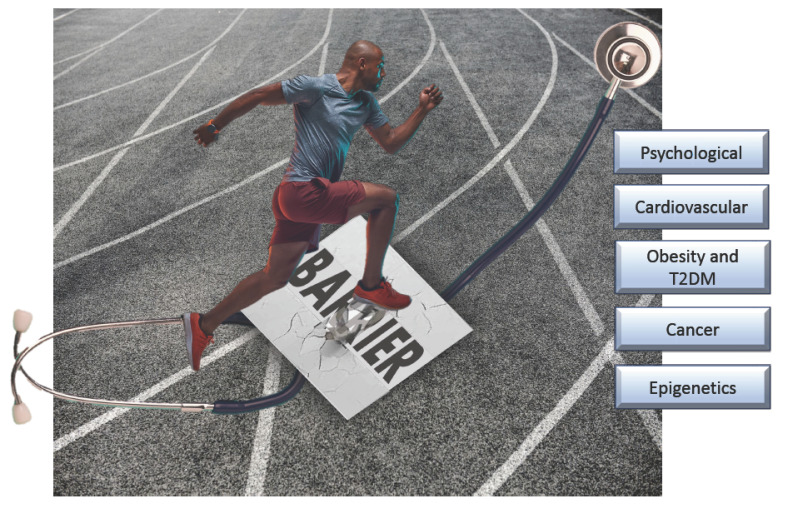
Exercise has many beneficial effects, including but not limited to prevention of and improvement in psychological, cardiovascular, obesity and type 2 diabetes mellitus (T2DM), cancer, and epigenetics disease states and outcomes, respectively. Despite the known beneficial effects of exercise, many barriers still exist in implementing exercise and physical activity in clinical practice for disease prevention and treatment. These barriers and how to reduce them deserve more attention in providing holistic, evidence-based best practices in patient care.

It is clear that exercise training, both aerobic and anaerobic, has far-reaching beneficial effects on health and disease prevention and treatment. Clinical guidelines are available for the prescription of physical activity for Americans, yet these guidelines are lacking in sufficient detail for specific recommendations for various populations using various modalities of exercise due to the limitations of current scientific research that has investigated the specific benefits of exercise modality, intensity, duration, etc., with respect to disease states in different age groups and populations to determine the best approach to using exercise training for disease prevention and treatment. In addition, throughout most of the US, where chronic diseases that can be easily prevented or treated with exercise cost $4.1 trillion in healthcare [71,75], the infrastructure to support more healthy physical activity is lacking. An easily accomplished first step to building this infrastructure is to utilize a more comprehensive approach to healthcare. One can easily envision an approach that can be adopted where general practitioners, such as family medicine physicians, are at the forefront of coordinating and helping patients implement dietary and exercise interventions for optimal health. In order for physicians to do this, support and buy-in from local and state agencies is required. Something as simple as building more sidewalks in neighborhoods and along streets, building and maintaining safe parks and recreational facilities that are “free” to the public, integrating biking paths that are safe to travel along streets, even incorporating gyms in local physician practices whose membership fees are covered by health insurance are some of the possible solutions that can start to provide the care needed to protect and improve the health of all Americans (Figure 1).

## Data Availability

No new data were created or analyzed in this study. Data sharing is not applicable to this article.
